# Factors that Predict Parental Willingness to Have Their Children Vaccinated against HPV in a Country with Low HPV Vaccination Coverage

**DOI:** 10.3390/ijerph15040645

**Published:** 2018-03-31

**Authors:** Maria Ganczak, Barbara Owsianka, Marcin Korzeń

**Affiliations:** 1 Department of Epidemiology and Management, Faculty of Medical Sciences, Pomeranian Medical University, Zolnierska 48, 71-210 Szczecin, Poland; 2 Multispecialty Hospital, Lubanska 11-12, 59-900 Zgorzelec, Poland; barbara10201@wp.pl; 3 Department of Methods of Artificial Intelligence and Applied Mathematics, Faculty of Computer Science and Information Technology, West Pomeranian University of Technology, Zolnierska 46, 71-210 Szczecin, Poland; mkorzen@wi.zut.edu.pl

**Keywords:** HPV, vaccination, awareness, attitudes, knowledge, determinants, parents

## Abstract

*Background:* Adolescent HPV (Human Papilloma Virus) vaccination is yet to be introduced as a mandatory program in Poland. Polish literature on factors associated with adolescent HPV vaccination is scant, despite the fact that uptake is one of the poorest in the European Union. *Objectives:* To assess HPV awareness and identify independent predictors for parental willingness to have their children vaccinated against HPV. *Methods:* All parents of first grade students from three selected high schools in Zgorzelec, Poland, who participated in parent–teacher meetings at the time the study was conducted, had their children unvaccinated regarding HPV, and who gave informed consent to participate were included. There were 600 first grade students; 9 were vaccinated against HPV. This left 591 parents who met the eligibility criteria; the response rate was 76.1%. *Results:* Awareness of HPV was reported by 55.3% of 450 parents (mean age 42 years, 70.9% females); 85.1% expressed their willingness to vaccinate their children against HPV; 31.3% identified HPV as a sexually transmitted pathogen, and 36.2% identified it as a risk factor of cervical cancer. Multivariable logistic regression analyses indicated that being employed (OR 2.09; 95% CI: 1.10–3.86), having positive attitudes toward vaccines (OR 3.02; 95% CI: 1.34–6.49), previous information about HPV (OR 2.02; 95% CI: 1.17–3.51), and concerns about the side effects of the HPV vaccine (OR 0.60; 95% CI: 0.35–0.99) were independent predictors of parents’ willingness to vaccinate. *Conclusions:* Attitudes regarding their child being vaccinated against HPV were positive among Polish parents, even though awareness and knowledge of HPV in this group were low. Most of the significant factors that influenced their willingness were modifiable, such as being informed about HPV and having positive attitudes toward vaccines. Future interventions should focus specifically on vulnerable subgroups, such as unemployed parents.

## 1. Introduction

Worldwide, an estimated 630 million persons are infected with human papillomavirus (HPV) [1]. The prevalence of HPV infections peaks in adolescence in both genders and increases every year from 14 to 24 years of age [2]; it is estimated that approximately one-quarter of HPV infections are acquired by adolescents [3]. Most of these infections are transient and asymptomatic. However, some HPV types cause warts on the skin or around the genital area and several—in particular, HPV 16 and HPV 18, so-called high-risk HPVs—can lead to high-grade lesions and eventually to HPV-associated cancers [4]. It is estimated that about 530,000 new cases of invasive cervical cancer were diagnosed worldwide in 2013, and an estimated 270,000 women died of this disease [4]. Poland has a medium incidence of and mortality from cervical cancer among European countries, with age standardized rates of 15.3/100,000 and 7.4/100,000, respectively, in 2012 [5]. Currently, every year in Poland, around 3500 women are diagnosed and around 1800 die as a result of this neoplasm [5].

A number of health organizations and scientific societies, e.g., the WHO (World Health Organization), the CDC (Centers for Disease Control and Prevention), the ECDC (European Centers for Disease Prevention and Control), and the AAP (American Academy of Paediatrics), recommend HPV vaccination for girls aged 11–12 years and a catch-up vaccination for those aged from 13 to 18 regardless of sexual activity, as well as persons who are not yet sexually active [6,7]. In recent years a number of countries have rolled out national HPV vaccination programs for girls, and, more recently, boys [4,7,8]. Each country varies in its approach to coverage, ranging from a small percentage to over 90% [7,9]. 

Within the EU, Poland is one of eight countries where HPV vaccination is yet to have been made part of a free-of-charge mandatory form of immunization programme. Vaccinations must be purchased at primary health care sites. Three vaccines—Silgard, Cervarix, and Gardasil 9—are licensed in Poland for the prevention of cervical and other genital neoplasia as well as genital warts; the cost of a full series is high (ca. 300–440 USD per series) [9]. HPV vaccination coverage in adolescent girls is estimated at 1.5–10% and is much lower than those reported in countries where the cost of vaccinations is covered from the national budget [9,10,11].

Over the last decade a variety of studies have explored adolescents’ and parents’ attitudes towards HPV vaccination, as well as the determinants of HPV vaccination uptake among adolescents in countries where the coverage in this subgroup is optimal or suboptimal [12,13,14,15,16]. Previous studies have identified factors associated with HPV vaccination decision-making including demographics, knowledge, attitudes, and social norms. The evidence has indicated statistically significant association between parental educational status, previous information about HPV, general belief in the protection offered by vaccination, receiving a healthcare professional’s recommendation, and HPV vaccine acceptance [15,16,17]. Belief that one’s child is too young to get vaccinated; concern about vaccine adverse effects, safety, and newness; and concern about the effect of vaccination on their child’s sexual behavior were reported as potential barriers [15,16,17,18]. Polish literature on factors associated with adolescent HPV vaccination is scant, despite the fact that uptake is one of the poorest in the EU [9,10,11,19].

## 2. Objective

The decision to vaccinate against HPV is often made by the parents and is affected by their knowledge of the vaccine and attitudes about its use [18]. Therefore, the study objective was to assess parental awareness of HPV and to identify factors associated with the decision to let their adolescent children receive the HPV immunization. Such evidence could be used to address and tackle the possible barriers to successful coverage of the HPV vaccine and work on optimal strategies to improve vaccination uptake in this at-risk population. A clear awareness of parent views regarding HPV immunization would enable better shaping of interventions that would, in turn, increase coverage. 

## 3. Materials and Methods

### 3.1. Design and Setting

A cross-sectional study was conducted from June 2013 to October 2014 among parents of high school students from the city of Zgorzelec with 32,332 inhabitants, located in southwestern Poland. 

### 3.2. Study Population and Sampling

At the time the study was conducted there were two phases of secondary education in Poland: lower secondary education (junior secondary education, gymnasium) for pupils aged 13 to 16 and upper secondary education (high school) for students aged 17 to 19. The study participants were parents of first grade high school students from three high schools in the city which comprised all high schools in this area. There were 22 first grade classes in the selected schools, and each class consisted of 10–34 students. All parents of the abovementioned students who participated in parent–teacher meetings at the time the study was conducted, had their children unvaccinated regarding HPV, and who gave informed consent to participate were included. Parents who were not present during the first parent–teacher meeting were asked to complete an anonymous questionnaire during the following meetings.

The larger study involved analytical survey of parents before and after a school-based intervention program targeting parents and comprised two parts:

1. preintervention (an assessment of parents’ knowledge about HPV, the willingness to vaccinate their adolescent child against HPV, and factors associated with such willingness);
2. the immediate postintervention phase (evaluation of immediate changes in HPV knowledge/a willingness to vaccinate their adolescent child against HPV after a short educational intervention).

The intervention used in parent–teacher meetings at schools consisted of a 30–40 min presentation about HPV and leaflets/brochures about HPV. 

This paper describes the results obtained from the first part of the study.

### 3.3. Study Instrument and Data Collection

A structured anonymous questionnaire was used as the main data collection instrument. It was designed by the study team using a literature review [12,13,14,15,16,17,18]. It was pilot-tested on 30 parents at one randomly selected class from one of the three participating high schools (results included in the study). Questionnaires were distributed to the parents via the respective schools during parent–teacher meetings and were administered by one of the researchers (Barbara Owsianka) with the help of teachers. Meetings were attended mostly by one parent. In those uncommon situations when both parents attended, one parent was randomly selected and then asked to fill out the questionnaire. Each questionnaire consisted of 30 multiple-choice questions with the possibility to choose one/more predefined answers. The information obtained from the questionnaires included (1) socio-demographic data, and personal and family history of cancer; (2) previous information about HPV and sources of information; (3) knowledge about HPV; (4) general attitudes towards immunization; (5) concerns about HPV infection; (6) willingness to vaccinate an adolescent child against HPV. 

Regarding HPV knowledge, eight items were rated as “true”, “false”, “don’t know”, with one point being given for a correct response. Questions were divided into three groups: symptoms/outcomes; transmission routes and risks; therapeutic methods. A scale was created to measure knowledge level from 0 to 8. A total knowledge score was then calculated for each respondent. HPV knowledge scores were set as follows: poor, 0–4 (less than 51% correct); adequate, 5–6 (51–75% correct); good, 7–8 (>75% of correct).

The study was approved by the local Ethic Committee in Wrocław (Komisja Bioetyczna przy Dolnośląskiej Izbie Lekarskiej, Uchwała nr 1/DR/2013).

### 3.4. Statistical Analysis

Categorical data were presented as frequencies with percentages and continuous data as means. Chi square test was used for two group comparisons of categorical variables; associations between continuous variables were assessed with the Mann-Whitney test. *p* values < 0.05 were considered statistically significant.

The variables of interest were as follows: age (≤42 years/>42 years), gender (male/female), place of residence (urban/rural), education level (basic–secondary/high), marital status (single/married–cohabitate), religion (religious/not religious), employment status (working: employed, homemaker/ not working: retired-disabled-unemployed), number of children in the family (≤2/>2), together with a cancer history in a family member/friend (yes/no-cannot recall), received information about HPV (yes/no), knowledge about HPV and attitudes toward vaccines such as: vaccines are effective in disease prevention (yes/no-do not know), concern the child is at risk regarding serious consequences of HPV infection (yes/no-do not know), concern that HPV vaccination would make their children more likely to have sex (yes/no-do not know), and concern about the side effects of HPV vaccine (yes/no-do not know), associated with an outcome variable. The question “Are you willing to vaccinate your child with HPV vaccine?” was used to assess the potential willingness to vaccinate a child with HPV vaccine. Several multivariable logistic regression models were constructed using the stepwise backward elimination method to arrive at the most parsimonious model with independent predictors for parental willingness to vaccinate. Initially, all explanatory variables listed above were considered in the models, regardless of an association or a trend toward an association shown with the outcome of interest in the biivariate analysis. The Akaike Information Criterion (AIC) was used to find the model of the best fit [20]. Regression results were presented as odds ratios (ORs) and 95% confidence intervals (CIs) for ORs. The quality of the model was assessed using deviance residuals, pseudo R2 coefficient, and Area under the Curve (AUC) analysis [21]. STATISTI-CA PL, Version 12.5 (StatSoft, Kraków, Poland) and R software (R Foundation for Statistical Computing, Vienna, Austria) were used for data analyses [22].

## 4. Results

There were 600 first grade students in the studied high schools at the time the study was conducted; 9 were vaccinated against HPV. This left 591 parents who met the eligibility criteria; the response rate was 76.1%.

### 4.1. Sociodemographic Characteristics

The sociodemographic characteristics of the participants are presented in Table 1. Of the 450 parents, the mean age was 42 years (range: 29–67 years), the majority were females (70.9%), almost two-thirds lived in urban areas (64.0%), and the majority (84.4%) had final education levels of high school (40.0%) or below (44.4%); more than three-fourths were currently working (76.7%), while the rest were not (16.4% were unemployed, 4.9% disabled, 2.0% retired). About three-fourths of participants (74.2%) were married or lived in partnership; almost a half (46.0%) had two children, 27.8% had more than two children, and 26.2% had one child (median 2; range: 1–6). The vast majority of parents declared they were religious (97.1%); among them, 92.2% were Catholics. One in seventeen participants (6.0%) had a personal cancer history, and half (49.8%) had a family/close friend with a history of cancer. Of 450 respondents, 233 (51.8%) were parents of first grade high school boys, and 217 (48.2%) of adolescent girls from the first grade. 

### 4.2. Sources of HPV Knowledge

Almost half of the parents (201; 44.7%) had never heard of HPV. The most common sources of HPV knowledge were television (53.8%), internet (41.4%), and leaflets (32.4%), followed by a gynecologist (25.2%), a pediatrician (11.4%), and a nurse (11%); this was a multiple-choice question.

### 4.3. Knowledge about HPV

The parental knowledge regarding HPV is presented in Table 2. The mean score of HPV knowledge was 2.73 (SD = 2.35). One-fifth (20.2%) of the respondents scored >50% of the correct answers. Regarding knowledge that HPV infection is always asymptomatic, about one fifth (19.1%) of respondents gave the correct answer. Only one in eleven parents (9.3%) correctly recognized that HPV infection does not always result in a clinical manifestation, such as genital warts. Only 36.2% of participants knew about the possible clinical outcomes of HPV infection and correctly selected HPV as a risk factor for cervical cancer; 27.6% correctly stated that HPV infection does not lead to AIDS. When asked about routes of HPV transmission about one-third of parents (31.3%) correctly identified sexual intercourse; 42.7% recognized that having multiple partners increases the risk of contracting HPV. However, 21.8% of parents stated that HPV infection is limited to females. In the overall sample, 14.0% correctly stated that HPV infection cannot be treated by antibiotics. There was no statistically significant difference in the knowledge level by parents’ education level (chi square test: 36/200 vs 55/250; *p <* 0.05).

### 4.4. Parents’ Attitudes and Willingness Regarding Vaccinating Their Children

Regarding parents’ attitudes toward HPV, 414 (92.0%) stated that vaccines are effective in disease prevention; 194 (43.1%) were concerned about possible side effects of vaccination, and 256 (56.9%) were not; 155 (34.4%) were concerned that vaccination would make their children more likely to have sex, 93 (20.7%) were not, and 202 (44.9%) were not sure; 169 (37.6%) considered their child as being at risk regarding serious consequences of HPV infection, 76 (16.9%) did not, and 205 (45.5%) were not sure.

Regarding parental willingness to vaccinate their adolescent children against HPV, 383 (85.1%) were willing to have their children undergo vaccination, 19 (4.2%) had a negative attitude, and 48 (10.7%) were not sure. In the opinion of the parents, the best age to vaccinate a child for HPV would be as follows: “15 years or more” (155; 34.4%), “12–14 years” (*n =* 178; 39.6%), “11 and less years” (*n =* 98; 21.8%); 19 parents (4.2%) stated “I do not know”. 

Parents were asked who, in their opinion, would have the most significant influence concerning their decision-making regarding child/children HPV immunization. Two of three participants (300; 66.7%) reported that any decision would be significantly influenced by a physician’s recommendation, almost one-third (142; 31.5%) would be their own sovereign decision, and 8 participants would possibly base their decision on recommendations made by family/friends.

### 4.5. Factors Influencing Willingness for Vaccination

Bivariate analyses revealed that more mothers and parents who were employed were willing to vaccinate their child for HPV (90.8% vs. 82.8%, *p =* 0.03 and 87.0% vs. 79.0%, *p <* 0.05, respectively) (Table 1). The rates of parents willing to have their children receive the HPV vaccine were higher in the group which believed that vaccines are effective in disease prevention (86.7% vs. 66.7%; *p =* 0.003) and those who had ever heard about HPV (88.8% vs. 80.6%; *p =* 0.02). The percentage of parents willing to vaccinate their child for HPV was significantly lower in the group concerned about the safety of HPV vaccine (80.9% vs. 88.3%; *p =* 0.03). 

As presented in Table 1, no differences were found in terms of age, residency, education level, marital status, number of children in the family, religiosity, family history of cancer, knowledge of HPV, concern that HPV vaccination would make children more likely to have sex, and concern that a child is at risk regarding serious consequences of HPV infection. 

Regression analyses were performed to assess factors associated with parent willingness to vaccinate adolescents against HPV (Table 3). Acceptance was higher among those employed (OR 2.09, *p =* 0.03), who had positive attitudes toward vaccines (OR 3.02; *p =* 0.006), and who had ever heard of HPV (OR 2.02, *p =* 0.01). Concerns about the possible side effects of HPV vaccine were associated with lower willingness (OR 0.60, *p <* 0.05) to have a child vaccinated. Of note, although one variable (age) was an insignificant (Wald’s test) predictor, it was still selected by a stepwise procedure and remained in the model together with some other, significant variables.

## 5. Discussion

### 5.1. Results Overview

Awareness of HPV among the parents of the first-year high school students was low. The most common sources of their knowledge regarding HPV were television, internet, and leaflets. The overall knowledge about HPV was poor: only about one-third of parents identified HPV as a sexually transmitted pathogen or as a risk factor of cervical cancer. Nevertheless, the majority intended to have their children undergo vaccination against HPV. In multivariable modelling, parents who were employed, had positive attitudes toward vaccines, and had ever heard of HPV were more likely to have their children vaccinated. Those who expressed concerns about HPV vaccine safety were less willing to vaccinate their children.

### 5.2. Awareness of HPV Infection

The awareness of HPV infection among parents is crucial as it may support vaccination in the case of their own adolescent children [12,14,16]. However, only 55.3% of parents in the present study had heard of HPV. This result is concordant with the results from a study conducted in the United Arab Emirates (UAE) among parents with teenage daughters [14], but is much higher than results reported in a study from China (19.3%) [12]. However, HPV vaccine had not been licensed in China at the time the study was conducted and HPV-related health promotion was inadequate. The present study and a UAE study, where higher levels of HPV awareness were observed, were carried out after HPV vaccine was introduced nationwide.

### 5.3. Knowledge about HPV

This report presents one of the few studies undertaken to evaluate the determinants of a willingness to vaccinate adolescent children against HPV that specifically considers HPV knowledge level within its analyses. Our results show a disturbingly low HPV knowledge level among parents of adolescent students. Only 19% of the studied population correctly stated that HPV infection may be asymptomatic. Almost two-thirds did not recognize HPV as a risk factor for cervical cancer, which is a much higher percentage than reported in the Chinese study (52.5%) but lower than the rate observed in the UAE (85.5%) [12,14]. This might be a source of concern, as a lack of knowledge about the possible severe clinical outcomes of HPV infection can negatively influence preventive measures, such as vaccination, and be attributed to a rise in cervical cancer incidence. In many studies [23,24,25], perceived risk of HPV-related disease was a predictor of vaccine acceptance and series initiation. In the present study, more than two-thirds of parents did not identify sexual intercourse as an HPV transmission route and more than half did not recognize that having multiple partners increases the risk of contracting HPV. This was also observed by others [14]. Since it is clear that HPV vaccine prevents sexually transmitted diseases, it may be speculated that the parents’ lack of knowledge regarding the HPV transmission route may negatively influence their decision to vaccinate a child before sexual debut [16]. Some myths still existed among studied population, such as HPV infection being only limited to females. Such a misperception could limit the willingness of parents to vaccinate their sons against HPV.

The low awareness and poor knowledge regarding HPV observed in our study population may be associated with a number of factors. Firstly, the demographic characteristics of this study sample showed that 44.4% only had primary education. Although there was no difference in the knowledge level by parents’ education level observed in this study, results from some previous studies show that higher education among parents was significantly associated with a higher level of knowledge regarding HPV [12,14,26]. Parents with a better education background may have more access to health information. Secondly, television, internet, and leaflets, not medical staff, were the main sources of the parents’ information on HPV. This may include incomplete or incorrect information.

### 5.4. Willingness to Immunize a Child against HPV

Our results indicate that parental attitudes toward vaccinating adolescent children against HPV were generally favorable. There are a number of previous surveys that examined this issue regarding both genders or exclusively daughters [12,13,14,15,17,27]. These studies revealed that support for the HPV vaccine was high, up to 74%, 75%, and 77% in Canada, the US, and the UAE, respectively [14,15,27]. Consistent with these prior studies, 85% of our respondents expressed willingness to have their children undergo vaccination, even though only 56% of them had heard of HPV. 

Adolescents included in this study belonged to the first grade of a high school, being at the age of about 17 years. This age would make all the students eligible for the first dose of HPV vaccine (11–12 years). The observed mismatch between the actual, disturbingly poor vaccination uptake and an overwhelming majority expressing willingness to vaccinate seems confusing. One of the possible explanations of this finding could be a social desirability bias—the tendency of parents to answer questions in a manner that would be viewed favorably by teachers present in the classroom where the questionnaires were filled out. This could have resulted in over-reporting of “good behavior” (willingness to vaccinate an adolescent child against HPV) or under-reporting of “bad” or undesirable behavior, instead of choosing responses that are reflective of their true feelings [12,28]. Comparative studies on parental willingness to have their children vaccinated against HPV conducted outside the school setting would be of value to better assess this issue.

An interesting study was conducted in the US among mothers who were nurses; the majority intended to vaccinate a daughter if she was 16 to 18 years of age, about a half intended to do so if she was 9 to 12 years [29]. Contrary to this study, in our questionnaire this was a single-choice, not a multiple-choice, question. Our results showed that for 40% of parents, the best age to vaccinate a child for HPV would be 12–14 years of age; only 22% preferred 9–11 years. Both the US study and the present study results show that parents would prefer their children to be vaccinated later than recommended by the CDC and the ACIP [30]. According to medical literature, the young age of the child was reported as a common reason for refusing or delaying HPV vaccination, and older girls were more likely to be vaccinated than younger girls [4,15]. Borena et al. speculated that parents find it incomprehensible to vaccinate a child for an effect required several years later and postulated to consider lifting the vaccination age few years, though still before sexual debut [4]. According to our findings, even though the acceptance rate regarding HPV vaccination among Polish parents was high, raising the vaccination age of the child could increase uptake.

### 5.5. Factors Associated with the Decision to Let Adolescent Children Receive the HPV Immunization

Factors associated with parents’ anticipated uptake of HPV vaccination for their daughters and sons identified in this study are complex and similar to those reported by other countries [15,16,18]. We found that a willingness to vaccinate an adolescent child was strongly associated (OR 3.02) with the general belief in the protection offered by the vaccination, and it was the most important correlate of vaccine acceptability. This is consistent with some previous studies [15,16].

We also found that receiving information on HPV was associated with a two-times-higher chance of willingness to vaccinate a child. Our results support previous evidence related to this issue [15,18,25,31,32]. Notable, in the study conducted in Canada, although the proportion of parents who reported having received information on the vaccine from their physicians was low, these parents were significantly more likely to allow a child to receive the vaccine than those who had other sources of information [15]. Two-thirds of parents surveyed in the present study reported that their decision on vaccinating their adolescent child would be significantly influenced by a physician’s recommendation. This is an important finding, which confirms that receiving a doctor’s advice or discussing the HPV vaccine with a doctor is associated with vaccine acceptance and initiation.

In our study, 43.1% of parents were concerned about HPV vaccine safety. Multivariable regression analyses revealed that it was a negative determinant of parental willingness to vaccinate a child against HPV. This may be explained by the fact that our study population received information mainly from the mass media, which tend to report predominantly on speculated negative outcomes of vaccines in general and the HPV vaccine in particular. This result is in line with current literature [12,14,15,16] which reported HPV vaccine side effects as the most commonly stated reason for not having a child receive the vaccine. Since the safety of the vaccine has been well documented in numerous randomized control trials [14,33,34], it is only the matter of improving communication tools which would raise parent awareness regarding this issue.

The present study did not identify a statistically significant association between parental HPV knowledge level and vaccine acceptance. This result is similar to findings reported in a US study [27] and a study conducted in India which found that while parents had limited knowledge of HPV or cervical cancer, most were still highly accepting of an HPV vaccine [13]. Other studies indicated low HPV knowledge level as a barrier [12,35]. The possible explanation of our results could be that almost half of the studied population had concerns about the vaccine’s potential side effects. Thus, it is possible that those who had greater HPV knowledge might also be more concerned about potential adverse events of the vaccine and were therefore more reluctant to vaccinate their children against HPV [34]. Further studies on a regional or national level are needed to better assess this issue.

### 5.6. Implications for HPV Immunization Policy

HPV vaccine acceptance in this study was associated with parental employment status, and employed parents were two times more likely to vaccinate their children. Governmental support that would make HPV vaccinations free of charge or partly subsidized would greatly increase uptake, especially among unemployed parents and/or those living on low income. Interventions should be initially directed to those areas that suffer from especially high incidence rates of cervical cancer [9].

Our study revealed that parents were not confident regarding the safety of the HPV vaccine. Therefore, interventions should concentrate on strategies which could increase confidence. Parents should be informed that it is a cancer-prevention vaccine, which can offer substantial benefits and might reduce the likelihood of serious outcomes [36]. The wider adoption of the Gardasil 9 vaccine (which prevents both low-risk HPV subtypes 6 and 11, which can cause genital warts, and high-risk HPV subtypes 16, 18, 31, 33, 45, 52, and 58, causing cervical dysplasia and cancer) in immunization programs, promises to increase vaccine effectiveness, which may in turn restore confidence [3,9,33]. Advances in vaccine technology which could possibly reduce the number of required doses to just one may additionally increase its use.

The results of the study also showed that being informed about HPV was associated with greater odds of parental willingness to immunize their child. Although healthcare providers play an influential role in parents’ decisions to vaccinate their children against HPV, the results of the study show that providers are not yet fully promoting the vaccine. Therefore, gynecologists, pediatricians, and GPs should increase their role in the communication and delivery of vaccination for this specifically vulnerable group at visits made by adolescents and their parents.

Clinicians should also consider informing the parents directly through messages by telephone, text, letter, postcard, or other media. Another tool used to provide information about HPV for the parents of adolescent children in Poland could be education by qualified experts during periodic parent–teacher meetings. As an example, Canada has begun to shift its attention to education funding for parents with regard to the prevention of HPV infections [37]. Additionally, media campaigns aimed at motivating parents to vaccinate their children against HPV could also be used. Such campaigns are effectively employed in a number of countries worldwide, such as the US [38].

## 6. Limitations

The study has a number of limitations. It was conducted in a small city; therefore, the findings do not necessarily apply to other students in the region or even the country, and further studies at national level would be of value. As mentioned in the Discussion section, there might also be response bias in the study: parents filled out the questionnaires in the presence of teachers, and thus may have given more sociably desirable responses [12,28]. However, the fact that the questionnaires were completed anonymously should result in reducing this bias. Ideally, all students’ parents (both vaccinated and nonvaccinated) should have been included in the study. Nevertheless, given that only 9 out of 600 were vaccinated, their inclusion/exclusion might not have altered the study results significantly. While we highlighted some variables which referred to parent demographics, other determinants, e.g., affordability of a vaccine, might have also influenced the willingness to vaccinate an adolescent child against HPV. Finally, the most important limitation of the evidence regarding the intention to vaccinate is that perceptions do not necessarily translate into vaccinations.

## 7. Conclusions

The surveyed parents obtained information regarding HPV mainly from nonprofessional sources which may influence their low knowledge level; however, the majority were in favor of their adolescent child being vaccinated against HPV. In common with other countries [14,15,18,19], there is not one single factor influencing parental support level regarding the HPV vaccination of their children in Poland, but rather a variety of factors, which may play different roles. Therefore, a systematic monitoring of parental attitudes towards HPV vaccination is needed to address adequate public health actions. Modifiable factors that influenced parental willingness regarding the HPV vaccine identified in this study are of public health interest as they offer an opportunity for experts in the field to design targeted interventions. There is a need for national education campaigns, together with advice and support from professionals at the community level, to increase HPV awareness and knowledge in terms of changing attitudes toward vaccination safety and to improve uptake. Future interventions should be more tailored, focusing especially on vulnerable subgroups identified in this study which are particularly difficult to reach, such as unemployed parents. Making HPV vaccines part of standard immunizations in Poland would be of immeasurable value. Finally, further quantitative but also qualitative studies on factors which affect parents’ or caregivers’ decision not to vaccinate children against HPV would be of value.

## Figures and Tables

**Table 1:** Characteristics of parents by a bivariate analysis of predictors of willingness to vaccinate an adolescent child against human papillomavirus (HPV) infection; Zgorzelec, Poland, 2016, *n =* 450.

Variable	Number (*n =* 450)	%	Willingness to Vaccinate (*n =* 383)	%	*p*
*Age*					
≤42 years	229	50.9	191	83.4	0.35
>42 years	221	49.1	192	86.9	
*Gender*					
Father	131	29.1	119	82.8	0.03
Mother	319	70.9	264	90.8	
*Residency*					
Rural	288	64.0	244	84.7	0.63
Urban	162	36.0	139	85.8	
*Education level*					
Primary education	200	44.4	163	81.5	0.06
Above primary education	250	45.6	220	88.0	
*Marital status*					
Married/Cohabitating	334	74.2	283	86.2	0.76
Single	116	25.8	100	84.7	
*Number of children*					
≤2	325	72.2	275	84.6	0.77
>2	125	27.8	108	86.4	
*Employment status*					
Working	345	76.7	300	87.0	0.05
Not working	105	23.3	83	79.0	
*Religious*					
Yes	437	97.1	372	85.1	1.0
No	13	2.9	11	84.6	
*Family history of cancer*					
Yes	224	49.8	189	84.4	0.66
No	226	50.2	194	85.8	
*Vaccines are effective in disease prevention*					
Yes	414	92.0	359	86.7	0.003
No/not sure	36	8.0	24	66.7	
*Concerned about the safety of HPV vaccine*					
Yes/not sure	194	43.1	157	80.9	0.03
No	256	56.9	226	88.3	
*HPV vaccination would make children more likely to have sex*					
Yes/not sure	295	65.6	246	83.4	0.17
No	155	34.4	137	88.4	
*My child is at risk regarding serious consequences of HPV infection*					
Yes	169	37.6	148	87.6	0.28
No/not sure	281	62.4	235	83.6	
*Had ever heard of HPV*					
Yes	249	55.3	221	88.8	0.02
No	201	44.7	162	80.6	
*Knowledge of HPV*					
Adequate/good	100	22.2	86	86.0	0.87
Poor	350	77.8	297	84.9	

**Table 2:** Parents’ knowledge about HPV; Zgorzelec, Poland, 2013/14, *n =* 450.

Statement	Correct Answer	True		False		Don’t Know	
		*n*	%	*n*	%	*n*	%
Having multiple partners increases the risk of contracting HPV	Yes	192	42.7%	14	3.1%	244	54.2%
HPV infection is limited to females	No	98	21.8%	55	12.2%	297	66.0%
HPV can be transferred via sexual route	Yes	141	31.3%	40	8.9%	269	59.8%
HPV is a risk factor for cervical cancer	Yes	163	36.2%	15	3.3%	272	60.4%
HPV infection is always symptomatic	No	59	13.1%	86	19.1%	305	67.8%
HPV infection can be treated by antibiotics	No	51	11.3%	63	14.0%	336	74.7%
HPV infection may lead to AIDS	No	24	5.3%	124	27.6%	302	67.1%
HPV infection always results in a clinical manifestation such as genital warts	No	45	10.0%	42	9.3%	363	80.7%

**Table 3:** Logistic regression model: association of parental willingness to immunize adolescents for HPV with variables selected with the use of a stepwise approach (OR’s estimates *, 95% Confidence Intervals of OR estimates), Poland, 2013–2016; *n =* 450.

Variable	OR	CI
Age: ≤42 years	1.50	0.77–4.78
Employment: yes	2.09	1.10–3.86
Positive attitudes toward vaccines	3.02	1.34–6.49
Had ever heard of HPV	2.02	1.17–3.51
Concerns about the side effects of HPV vaccine	0.60	0.35–0.99

* Odds ratio (OR) = ratio between the two categories tested in each variable, controlling for other variable.
